# Deep tuning of photo-thermoelectricity in topological surface states

**DOI:** 10.1038/s41598-020-73950-z

**Published:** 2020-10-07

**Authors:** Shouyuan Huang, Ireneusz Miotkowski, Yong P. Chen, Xianfan Xu

**Affiliations:** 1 School of Mechanical Engineering, Purdue University, West Lafayette, IN 47907 USA; 2 Birck Nanotechnology Center, Purdue University, West Lafayette, IN 47907 USA; 3 Department of Physics and Astronomy, Purdue University, West Lafayette, IN 47907 USA; 4 School of Electrical and Computer Engineering, Purdue University, West Lafayette, IN 47907 USA; 5 Purdue Quantum Science and Engineering Institute, Purdue University, West Lafayette, IN 47907 USA

**Keywords:** Nanoscience and technology, Physics

## Abstract

Three-dimensional topological insulators have been demonstrated in recent years, which possess intriguing gapless, spin-polarized Dirac states with linear dispersion only on the surface. The spin polarization of the topological surface states is also locked to its momentum, which allows controlling motion of electrons using optical helicity, i.e., circularly polarized light. The electrical and thermal transport can also be significantly tuned by the helicity-control of surface state electrons. Here, we report studies of photo-thermoelectric effect of the topological surface states in Bi_2_Te_2_Se thin films with large tunability using varied gate voltages and optical helicity. The Seebeck coefficient can be altered by more than five times compared to the case without spin injection. This deep tuning is originated from the optical helicity-induced photocurrent which is shown to be enhanced, reduced, turned off, and even inverted due to the change of the accessed band structures by electrical gating. The helicity-selected topological surface state thus has a large effect on thermoelectric transport, demonstrating great opportunities for realizing helicity control of optoelectronic and thermal devices.

## Introduction

Tetradymites (e.g., Bi_2_Te_3_ and Bi_2_Se_3_) are known as one of the best room-temperature thermoelectric materials for decades^1–4^, and have also been studied for infrared photodetectors and photovoltaics due to their narrow-bandgap and high mobility^5–7^. In recent years, they have also been investigated as three-dimensional topological insulators (3D TIs)^8–13^, a new phase of condensed matter possessing metallic states with gapless Dirac dispersion on the surface and gapped states in the bulk. In doped or ternary/quaternary tetradymites, the Fermi level can be optimized deep into the bandgap to suppress bulk contribution^14–16^. For example, it is shown that in sub-20-nm thin-film Bi_2_Te_2_Se and BiSbTeSe_2_, the topological surface states (TSS) dominate the electrical transport even at room temperature^15,17^. The TSS have spin-polarization locked to its momentum to prevent back-scattering^18,19^. The spin-momentum locking of TSS offers a chance to control the direction of the carrier flow, which is achieved using circularly polarized light that pumps the spin-momentum locked TSS asymmetrically in the k-space, leading to a measurable photocurrent^20–22^. This phenomenon is also called circular photogalvanic effect, and the generation of photocurrent does not require spatial inhomogeneity of either device structure or optical excitation.

For thermal transport, intriguing thermal transport properties of TSS have also been found in Bi_2_Te_2_Se thin films^23^. A much enhanced Lorenz number was observed, which is possibly originated from the unique momentum and energy relaxation processes of the TSS, i.e. carriers relax much faster in momentum transfer than in energy transfer due to strong electron interaction within the Dirac system, thus, charge and electronic thermal currents are decoupled, similar to what was observed in graphene^24^. The spin and energy relaxation dynamics has been shown by circularly-polarized pump-probe studies that spin depolarizes along with energy relaxation^25^. Direct demonstration of momentum relaxation processes within TSS is lacking and is expected to be different from the case when there is TSS—conduction band interaction^26^. Aside from direct measurements, it is known that momentum and energy relaxation times are related to electrical charge transport and thermal energy transport, respectively, and can be described equivalently by either Boltzmann transport equation or Landauer formalism^27–29^. The thermoelectric effects, including the Seebeck voltage under temperature gradient and the Peltier heat flow accompanied by the electrical current, involve both momentum and energy transfer processes and thus correlate with both relaxation dynamics. The thermoelectric properties of bulk and nanostructured tetradymites have been well-studied, however, the contribution from its surface states has yet been much investigated, and the attempts are limited to surface band-bending effects on thermoelectric properties in Bi_2_Te_3_ nanoplates^30^, bulk/surface two-channel analysis^31–34^, laser-heated in-plane heterostructures^35^, and photocurrent measurement in addition to the photothermoelectric current using circularly polarized light^36^. A relatively small (~ 15%) additional photo-response was observed compared to the photothermoelectric effect when the polarity of helical current matches with the thermoelectric current. It will be critical to understand the contribution of each type of carriers, e.g. TSS versus bulk. The thermoelectric properties can be tuned by electrical field-effect gating^15,19,30,37^ and temperature^38^ via effectively modulating the carrier densities of both TSS and bulk states, and the polarization-dependent photocurrent can be similarly tuned^22,37,39^. Thus, the combination of optical helicity and electrical gating can serve as a potent tool for deep tuning of photo-thermoelectricity in TSS.

In this work, we studied the photo-thermoelectric effect of TSS in 3D topological insulator Bi_2_Te_2_Se thin films. We demonstrated deep tuning of the Seebeck coefficient with the use of optical helicity and field effect at room temperature. This deep tuning is originated from the optical helicity-induced photocurrent, and a detailed study of the helicity-controlled photocurrent was carried out via back gating that explored the optical response at varied Fermi level. As the Fermi level moved across the bandgap, the helicity modulated Seebeck effect could be turned up, turned off, and even reverted due to optical injection of spin polarization to varied states of the band structure. The large effect of optical control of TSS on thermoelectric transport indicates the possibility of spin-selection and control of thermal transport in novel TI devices.

**Figure 1:**
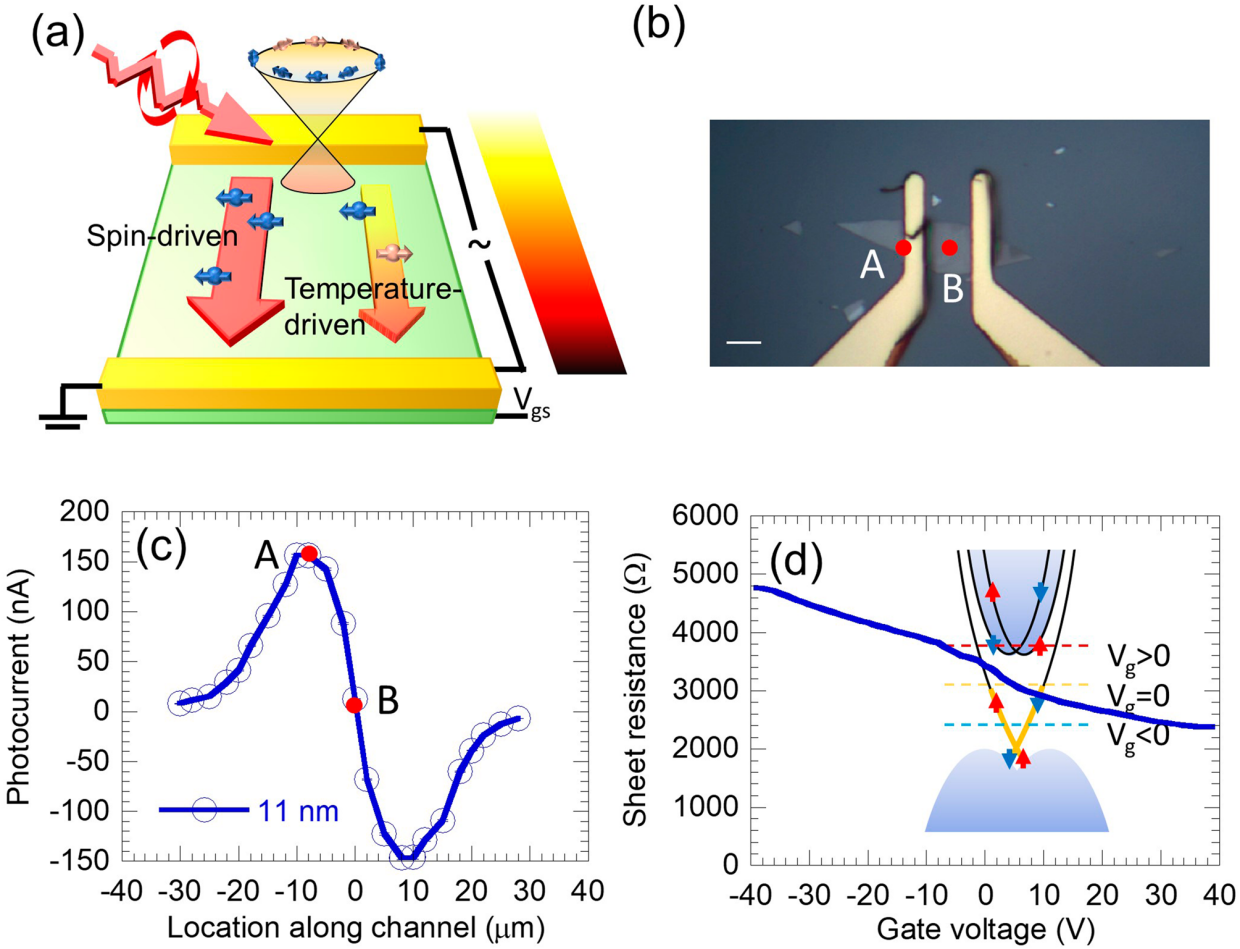
Experimental setup. (**a**) Schematics of the experimental setup; (**b**) Optical image of the 11-nm Bi_2_Te_2_Se device, red dots representing the laser focused at the contact (A) and at the center (B). Scale bar: 5 μm. (**c**) Photocurrent of incident light (polarized perpendicular to the current) focal spot located along the channel. Location A and B are also marked. (**d**) Gate-dependent sheet resistance of the 11-nm Bi_2_Te_2_Se device. Inset: Schematics of the band structure of the Bi_2_Te_2_Se topological insulator. Intrinsic Fermi level is within the bandgap and above the Dirac point, negative gating brings the Fermi level down to around but above the Dirac point, and positive gating raises the Fermi level up to accessing the bulk band/Rashba states.

Bi_2_Te_2_Se thin films are exfoliated from bulk crystal using dicing tapes and transferred onto SiO_2_/Si substrate, followed by fabrication of Cr/Au contacts by photolithography and e-beam evaporation metallization. A 1550-nm laser (below the bandgap of the silicon substrate to avoid additional photogating^40^) is focused (focal spot sized ~ 5 μm) onto the device using an off-axis parabolic mirror at a 45 degrees obliquely incident angle as shown in Fig. 1a. An optical image of an Bi_2_Te_2_Se device (thickness 11 nm) is shown in Fig. 1b. The laser beam is modulated by a mechanical chopper and the polarization is controlled by a quarter-waveplate (QWP). Laser polarization is perpendicular to the current at QWP angle α = 0 and converted to right/left-hand circular polarized at α = π/4 and 5π/4, and α = 3π/4 and 7π/4 respectively. A DC gate voltage is applied to the source contact and the back gate of highly doped silicon substrate across the substrate oxide (50-nm thickness SiO_2_). The photocurrent output across the source and drain contact is amplified by a current amplifier (the input impedance 50 Ω is much lower than the device resistance of a few kΩ) and then measured by a lock-in amplifier at the laser chopping frequency. This configuration is used for both photo-thermoelectric effect and helicity-controlled photocurrent measurements. All experiments are conducted at room temperature.

## Results and discussion

### Measurement of the Seebeck coefficient

The laser spot is first scanned across the channel of the 11-nm Bi_2_Te_2_Se device. The photocurrent pattern is shown in Fig. 1c. The positive and negative peaks of the current near the contacts and the reduced current in between indicate a photo-thermoelectric effect. The center of the channel can be found as the midpoint of the two peaks. Carriers are generated at the laser-heated hot spot then diffuse to both contacts. The current measured between the contacts is the difference between the diffuse currents received by the two contacts. The carrier type can be determined as n-type by the polarity of the current when light is focused near the source or the drain (left is source and right is drain in Fig. 1c). We also measured the sheet resistance of the device with different back gate voltages as shown in Fig. 1d. The decrease of the sheet resistance versus back-gate voltage also indicates the n-type carrier, and the monotonic decrease indicates that the Fermi level is always above the Dirac point at all back gate voltages used in the experiment as illustrated in the band structure shown in the inset of Fig. 1d^15,23^. (Absolute value of the Seebeck coefficient is used in the discussion below since the sample is always n-type) Our measurements also confirm Ohmic contact, since no signature of photodiode due to Schottky barriers is observed in the photocurrent scanning^41^. The peak of the current at the source contact (position A in Fig. 1b) is selected to extract the Seebeck coefficient for maximum signal.

The Seebeck coefficient (*S*) is extracted using the temperature rise and the short-circuit current by laser heating. The short-circuit Seebeck current is directly taken from the polarization-insensitive photocurrent term (details of the photocurrent will be given later). We measured thin-film Bi_2_Te_2_Se properties including optical properties^42^, electrical property (Fig. 1c, where the contribution of the contact resistance is typically < 300 Ω, and is thus neglected in the calculation), and thermal conductivity^23^, and then used a numerical model to find the temperature rise. The temperature rise is then coupled to a thermoelectricity model consisting of Seebeck voltage, Ohm current, Peltier heat flow, and Joule heating, to extract the Seebeck coefficient by matching the calculated short-circuit current with the measured value. In addition, we directly measured the Seebeck voltage and local temperature using micro-Raman thermometry under a 633-nm laser normal incidence to validate the Seebeck coefficient extraction. Details and validations of the method used for extracting the Seebeck coefficient are provided in Supplementary Note 1.

**Figure 2:**
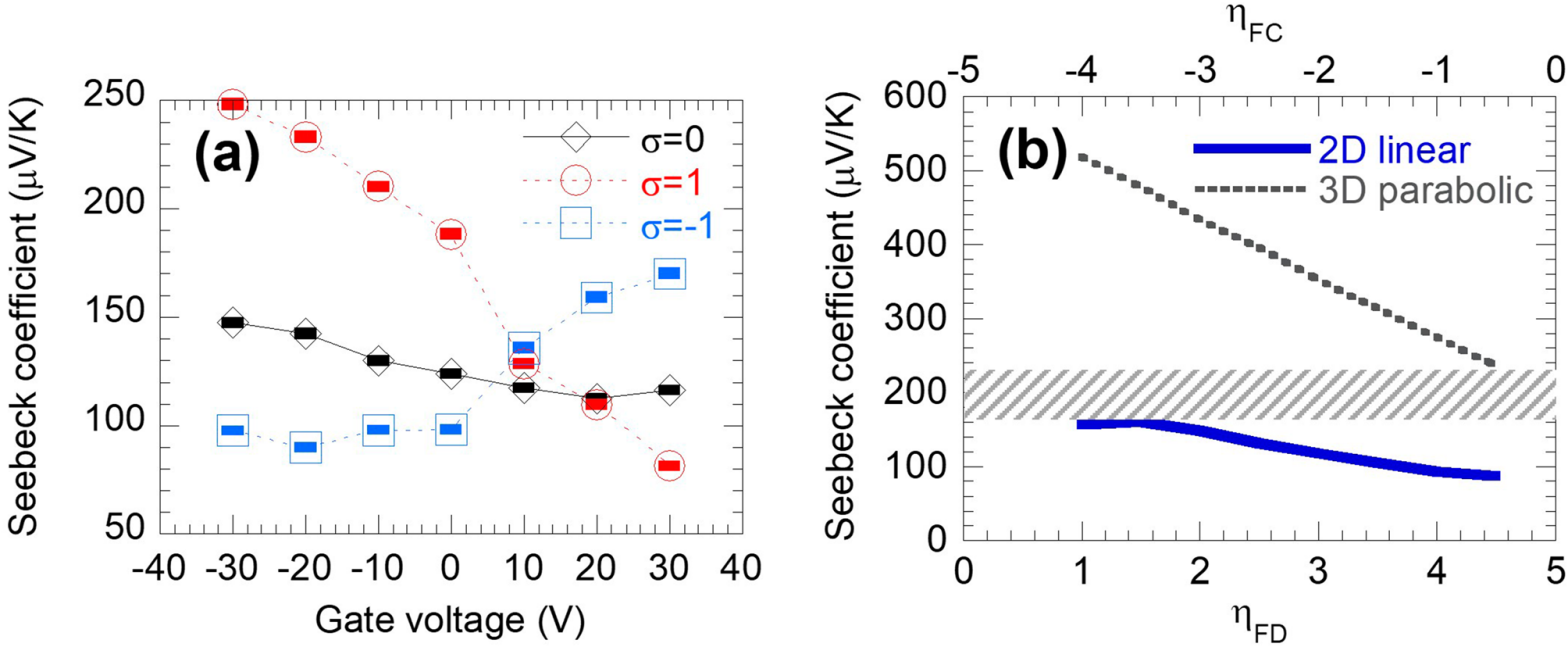
Gate-tunable thermoelectric performance of the 11-nm thin-film Bi_2_Te_2_Se device, with helicity controlling (**a**) (Absolute value of) Seebeck coefficient (*S*) measured under zero spin injection ($\upsigma$=0), and the extracted Seebeck coefficient using the total photocurrent measured under circularly polarized light ($\upsigma$= ± 1). (**b**) Seebeck coefficients of 2D linear dispersion (solid blue line) and 3D parabolic dispersion (dotted black line) electron systems versus normalized Fermi level calculated by the Landauer formalism.

We first measure the Seebeck coefficient resulted from the photo-thermoelectric effect using linearly-polarized light, hence, without spin injection, under different back-gate voltages. Our previous Hall effect measurements^23^ showed that the 2D carrier density of sub-20-nm Bi_2_Te_2_Se thin films is ~ 1 × 10^13^ cm^−2^, which is attributed to the TSS (5 × 10^12^ cm^−2^ each surface), and the bulk carrier concentration is less than 2 × 10^12^ cm^−2^. Estimation of Fermi level shifting and bulk depletion depth using the MOS capacitance model showed that when a 20 V gate voltage is applied, the depletion depth is ~ 32 nm (Supplementary Note 2). Thus, the entire film including the top surface of our 11-nm thin-film device can be effectively tuned by the back-gate. The measured Seebeck coefficient is plotted against the gate voltage in Fig. 2a (σ = 0). This Seebeck coefficient decreased from 147 μV/K at large (V_gs_ = − 30 V) negative gating to 112 μV/K when Fermi level moved towards the conduction band, then slightly increased to 116 μV/K (V_gs_ = − 30 V). The non-monotonic relation of the Seebeck coefficient and Fermi level indicates an interplay of different types of carriers.

The measured Seebeck coefficient mainly originates from the TSS carriers. This origin of the thermoelectric effect can be analyzed using the Landauer formalism. Unlike electrical conductivity or electronic thermal conductivity which depends much on the exact band structure, the Seebeck coefficient is only weakly affected by the details of band-structure and effective mass. For example, for all n-type bulk semiconductors with a parabolic band, the Seebeck coefficient versus Fermi level relation falls on the same curve despite the difference in effective mass^28,43^. Different Seebeck coefficient is only a result of different dimensionality (e.g., 2D electrons at the surface vs 3D electrons in the bulk) and the scattering rate, which can be calculated from the density of state. Hence the single band Seebeck coefficient of the TSS can be estimated using the Landauer formalism for 2D electron states with linear dispersion (details in Supplementary Note 3). Considering the possible Fermi level, the calculation results in Fig. 2b show that the Seebeck coefficient of 2D electrons is in the range 80–160 μV/K. (Normalized Fermi level, *η*_FD_ = (*E*_F_ − *E*_D_)/k_B_*T* for TSS w.r.t. the Dirac point *E*_D_, and *η*_FC_ = (*E*_F_ − *E*_C_)/k_B_*T* for conduction band w.r.t. its band edge *E*_C_, are used in Fig. 2b.) The measured Seebeck coefficient, ~ 120 μV/K, falls in this rage. On the other hand, the Seebeck coefficient of the parabolic 3D electrons in bulk is greater than 220 μV/K when the Fermi level is below the band edge, much larger than the measured Seebeck coefficient. Therefore, it can be concluded that in this thin film, TSS electrons are the dominant carrier for temperature-driven electrical transport. The large contribution from TSS to the Seebeck coefficient under laser heating is also recently reported in Ref.^38^. It has also been reported that under controlled Fermi level and scattering, TSS dominance could improve the power factor^33^, also, a TSS dominant conduction but bulk dominating Seebeck effect can be achieved^31,34^. Notice this discussion also explains the slight increase at very large positive gating, i.e. the conduction band kicked in as its carrier density increased.

### Photo-thermoelectrics in TSS with optical spin injection

Now we use circularly polarized incident light and repeat the photocurrent measurement to study the photo-thermoelectric effect with optical spin-injection. In addition to the optical heating, the right/left-handed circular polarization ($\upsigma$= ± 1) simultaneously inject up/down spin polarization to the TSS, so that the preferred motion of the excited TSS is parallel/antiparallel to the carrier flow driven by the temperature gradient, respectively, due to the spin-momentum locking. A same temperature rise is achieved when the incident light is circularly polarized, while the resulted electromotive force is tuned by the additional TSS current from spin injection. Thus we adopt the thermoelectricity framework here to present the result of carrier diffusion that is driven by the spin current in addition to the temperature gradient in terms of the thermoelectric property, i.e. an apparent Seebeck coefficient. The measured photocurrent is subsequently used to extract the apparent Seebeck coefficient using the model presented above, and is also plotted in Fig. 2a to compare with results without optical spin injection ($\upsigma$= 0). Under right-handed circular polarization ($\upsigma$= + 1), the extracted Seebeck coefficient rises to 248 μV/K when a 30 V negative gate voltage is applied, 60% more than that without spin injection under the same gate voltage (147 μV/K at V_gs_ = − 30 V). As the gate voltage is increased to positive, the Seebeck coefficient drops. Interestingly, the drop is much steeper than that when $\upsigma$= 0, down to 84 μV/K at V_gs_ =  + 30 V, below the value for $\upsigma$= 0 (116 μV/K). Hence, the optical spin injection broadens the tuning range of the Seebeck coefficient *S*_max_ − *S*_min_ by more than five times, or ratio *S*_max_/*S*_min_ by more than twice throughout the range of gate voltage. Similarly, under left-handed circular polarization ($\upsigma$= − 1), the optical spin injection of TSS that moves antiparallelly to the temperature-driven carrier flow suppresses the photo-thermoelectric current when a zero and negative gate voltage is applied, and enhances the photo-thermoelectric current under large positive gating. The apparent Seebeck coefficient of $\upsigma$= − 1 presents an opposite trend from that with zero and $\upsigma$= + 1 spin-injection, increasing from 90 to 170 μV/K with gate voltage, and the tuning range and the ratio are also much larger than the case without spin injection. It is also noticed that, when the Fermi level is deep into the bandgap (V_gs_ = − 30 V), the photo-thermoelectric current driven by the incident light with different polarization can differ by as much as five times. Hence, the combination of the optical helicity and electrical gating can significantly tune the photo-thermoelectric current in thin-film Bi_2_Te_2_Se devices, and this the large tunability of the Seebeck coefficient where TSS is dominant in thermoelectric transport offers a possibility of realizing thermal energy devices using helicity-controlled heat carriers.

### Gate controlled helical current in TSS

The large tunability of the Seebeck coefficient is originated from the large tunability of the helicity induced current. Here we describe our on the helicity induced current without the thermoelectric effect. We focus the laser spot at the center of the device (location B in Fig. 1b) where the photo-thermoelectric current towards the two contacts is balanced as seen in Fig. 1c. To study polarization-dependence, circularly polarized light is converted from linear polarized light using a QWP. The polarization-dependent photocurrent can be expressed as^22^:

1 $$\begin{array}{*{20}c} {j_{y} = D + C\sin 2\alpha + L_{2} \sin 4\alpha + L_{1} \cos 4\alpha } \\ \end{array}$$

where α is the angle between the incident linear polarization and the slow axis of the QWP. The magnitude of the circular component follows a sin2α form, i.e. the output light is right-hand circularly polarized when α = π/4 and 5π/4, left-hand circularly polarized when α = 3π/4 and 7π/4, and elliptically polarized in between. The coefficient *C* of sin2α represents the helical current of TSS. The TSS origin of the helicity-dependent photocurrent has been confirmed in tetradymite TIs via carrier modulation^37,39^ and ultrafast dynamics^44,45^. The sin4α and cos4α terms represent the current due to linear polarization rotation associated with QWP rotation, originated from the linear photogalvanic or photon drag effect^46,47^. The *D* term is polarization-insensitive, and is only attributed to thermoelectric effect due to optical heating (other effects are ruled out as discussed above). The *D* term at location A is the value we used to extract the Seebeck coefficient without spin injection.

**Figure 3:**
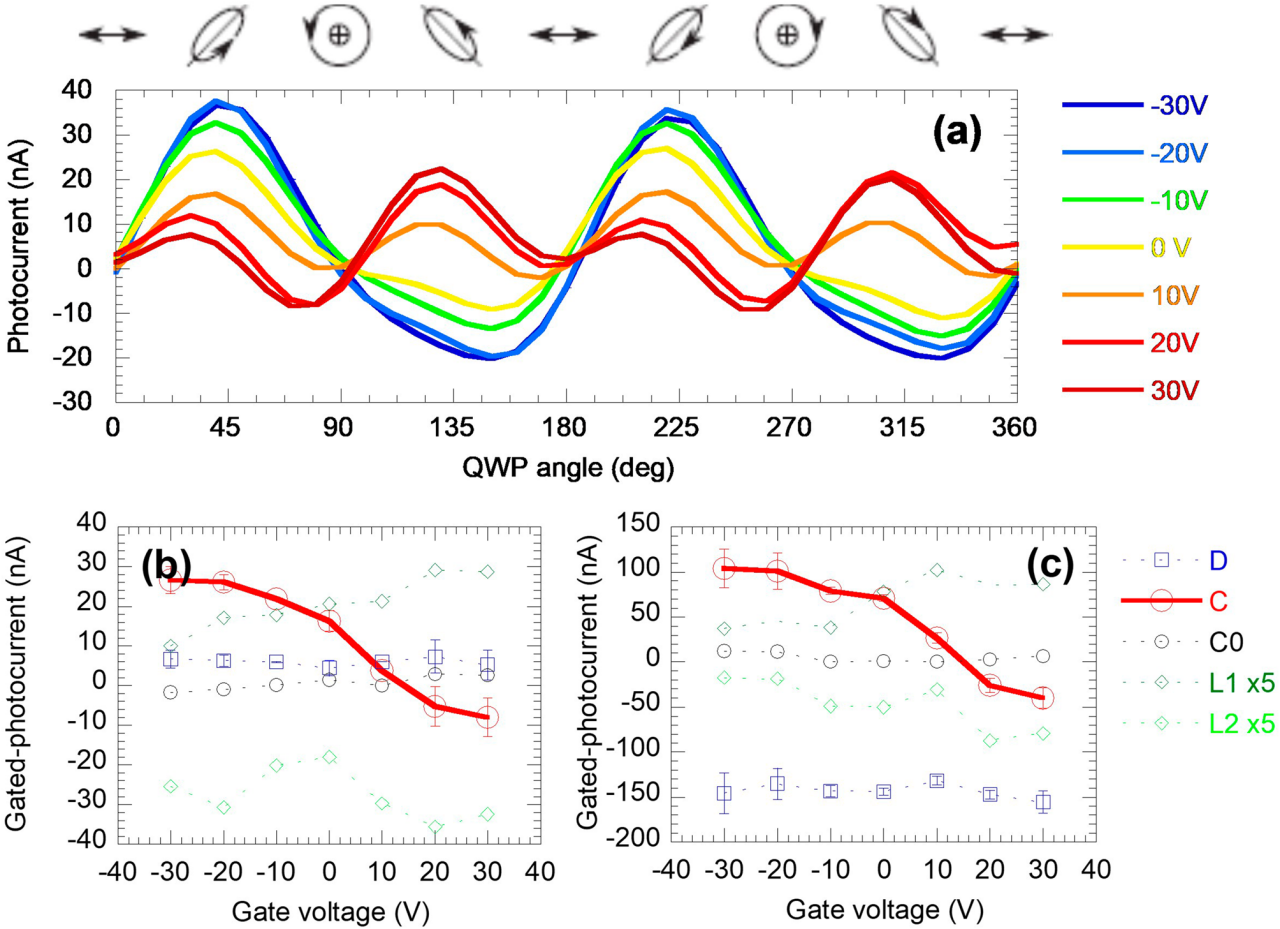
Photocurrent components of the 11-nm Bi_2_Te_2_Se device under back-gating. (**a**) Photocurrent versus quarter waveplate curves at varied gate voltage. The focal spot is located at the center of the channel. (**b**, **c**) Polarization-dependent components under varied gate voltage, with the focal spot at the center (**b**) and at the left contact (**c**). L_1_ and L_2_ are multiplied by a factor of 5 for clarity. D denotes the polarization-insensitive component of photocurrent; C is the circular-polarization-dependent photocurrent; L_1_, L_2_ are the circular-polarization-dependent photocurrent; C0 is the fitting coefficient of the cos2α term, i.e. artifacts due to measurement uncertainties.

Figure 3a shows that the photocurrent versus QWP angle evolves with the back-gate voltage when the Fermi level is tuned to access different parts of the electron band. At zero and negative gate voltage, a clear helicity dependent photocurrent is observed following the sin(2α) trend (Eq. 1), indicating the *C* component is dominant. This circular photocurrent is driven by the angular momentum selection rule of the spin-momentum locked topological surface states under helical incident light. (The current polarity is the same as the enhancement/reduction of the photo-thermoelectric effect and agrees with the spin selection of the optically injected angular momentum used in our experiment configuration.) The result is similar to earlier works^22,39,44^, while the magnitude of the helical current (both the absolute photo-response and the relative intensity of *C* to other components of the photocurrent *L*_1_ and *L*_2_) is much greater due to the longer spin lifetime^25^ when the Fermi level is within the bulk bandgap.

The fitted components of the polarization-dependent photocurrent are plotted against the gate voltage in Fig. 3b to show the field effect more clearly. Negative gating depletes electrons, and the Fermi level is tuned lower, away from the conduction band edge and towards the Dirac point (Fig. 1d inset), reducing both Pauli blocking by opening up more TSS available for excitation from the valence band^48,49^, also possibly reducing the scattering with spin-degenerated states in the conduction band that causes spin depolarization^50^. Both effects result in a larger photocurrent as shown in Fig. 3b. Despite the reduction of the density of states near the Dirac point, the photo-generated carrier density is not much affected due to the relatively low pumping rate of the incident light and the fact that the photon energy (~ 0.8 eV) is much larger than the bandgap (~ 0.3 eV). (Using the irradiation intensity and surface state absorption^42^, the carrier generation rate is estimated to be below 10^21^ cm^−2^ s^−1^ with the quantum efficiency not exceeding unity. Together with the ~ 10 ps carrier lifetime^25^, the excited carriers density is below 10^10^ cm^−2^, less than 0.2% of the carrier density on one surface. This excited carrier density also includes the valence band to conduction band transition. Hence the actual excitation density involving TSS is even smaller.) When a large enough positive gating is applied, we observe that the circular photocurrent is sharply reduced to zero and then flipped its sign. This is likely due to the spin-wise selection of the Rashba states created in the conduction band^50,51^. These states possess an opposite spin-texture to that of the TSS^22,50–52^ and thus resulting in a helical current in the opposite direction. Additionally, there is another possible transition that cannot be easily ruled out. A second topological surface state (TSS-2) with similar Dirac-like dispersion has been found in Bi_2_Se_3_, ~ 1.5 eV above its intrinsic Fermi level^26^. Bi_2_Te_2_Se is expected to share a similar band structure but the exact energy level of TSS-2 is yet to be studied. The transition from the conduction band to the TSS-2 below the Dirac cone, which has a negative group velocity, may also result in the inversed helical current.

The magnitudes of the sin/cos(4α) terms, denoted by *L*_1_ and *L*_2_, are also obtained by fitting the curves to Eq. (1), which are caused by linear photo-galvanic effect due to its dependence on the rotation of the linear polarization. Since the focus here is on the helicity dependent current, we provide a discussion on the origin of the observed linear photo-galvanic effect in Supplementary Note 4. From Fig. 3b we can also notice a slight increase of linear polarization-dependent photocurrent (the magnitude of *L*_1_, *L*_2_) with increasing positive gate voltage. This increase is due to the Fermi level approaching the conduction band. Conversely, under negative V_g_, the Fermi level recesses deeper into the bulk bandgap so the contribution from the topologically trivial states in the conduction band is reduced. The measured linear photo-galvanic components reduce to a finite number but not zero. This indicates that both topological surface state and the trivial state due to the band bending contribute to the linear-polarization-dependent photocurrent. A persisting linear-polarization-dependent photocurrent associated with the helical photocurrent is also observed in previous works^22,44^.

The gate-dependence of each component of photocurrent is also compared with the results when the laser focused at position A (Fig. 3c). The circular photocurrents exhibit almost the same trend of gate-dependence at both locations, indicating the conclusion obtained at the center of the device is also valid for that at the contact. Compared to the result at the center of the device, the helical current at the contact is nearly four times larger, which is due to the finite diffusion length of the spin. In fact, the ratio of the helical photocurrent magnitude at the center and the contact of the 6-μm channel can be used to estimate spin diffusion length L_s_, which turns out to be ~ 5 μm assuming an exponential decay of helical photocurrent magnitude versus distance (*x*) between the laser spot and the contact *C*_center_/*C*_contact_ = *C*(*x*)/*C*(0) = exp(−*x*/L_s_). This spin-diffusion length agrees with our previous ultrafast pump-probe measurement of the spin lifetime (a few 10 s of ps)^25^ and magneto-transport measurement of the Fermi velocity (1–2 × 10^5^ m/s)^14^, which give a spin diffusion length on the order of a few micrometers.

Thicker (27 nm and 74 nm) Bi_2_Te_2_Se devices are also studied using the same approach to further understand the mechanism when more bulk state carriers are involved as the films thickness is increased^15,23^. The Seebeck coefficients in these two devices are both above 300 μV/K, indicating the origin of the bulk states. The spin injection to TSS can still be observed. However, the combined spin injection and field effect on Seebeck coefficient tuning is much weaker than the 11-nm device. Details are provided in Supplementary Note 5.

## Conclusion

To conclude, our experiment showed highly effective helicity control of topological surface states under electrical field gating, and the contribution of the helicity-controlled TSS to electro-thermal transport. The helical photocurrent is amplified when the Fermi level is deep in the bandgap approaching the Dirac cone when Pauli blockage and spin-depolarizing scattering are reduced. On the other hand, the helical photocurrent is diminished when the Fermi level is close to the conduction band edge, and is even inverted when accessing the conduction band Rashba states. As a result, the photo-thermoelectric current can differ by as much as five times depending on the polarization of the heating laser, and the gate tunability of the photo-thermoelectric current can also be broadened by more than five times. These results showed the potential for effective manipulation of topological surface states and building helicity-controlled optoelectrical and thermal devices.

## Supplementary information

Supplementary Information.

## Abbreviations

3D: Three-dimensional
TI: Topological insulator
TSS: Topological surface state
QWP: Quarter waveplate
