# Supplementary material for: Deep tuning of photo-thermoelectricity in topological surface states
Source: Sci Rep. 2020 Oct 7;10:16761. doi: 10.1038/s41598-020-73950-z (PMC7541493; doi:10.1038/s41598-020-73950-z)
Supplement: Supplementary file 1 — Supplementary Information. [file 41598_2020_73950_MOESM1_ESM.docx]

Supplementary Notes

Deep tuning of photo-thermoelectricity in topological surface states

Shouyuan Huang^1,2^, Ireneusz Miotkowski^3^, Yong P. Chen^2,3,4,5^, Xianfan Xu^1,2*^

1 School of Mechanical Engineering, 2 Birck Nanotechnology Center, 3 Department of Physics & Astronomy, 4 School of Electrical and Computer Engineering, 5 Purdue Quantum Science and Engineering Institute, Purdue University, West Lafayette, Indiana 47907, USA

**Supplementary Note 1.** Validation of thermoelectric property extraction using optoelectrical and optothermal measurements

The Seebeck coefficient described in the main text is extracted by finding the temperature rise using a heat transfer model. During those measurements when there is laser irradiation at an oblique angle to generate photocurrent, the micro-Raman thermometry for temperature measurement (see below) cannot be applied due to limited spacing. To validate the Seebeck coefficient extraction procedure described in the main text, we measured the photo-thermoelectric (Seebeck) voltage with a direct local temperature rise measurement using micro-Raman thermometry at normal incidence^1^ (photocurrent is not measured during this validation). The Seebeck coefficient is calculated from the temperature and the measured Seebeck voltage using *V*_s_ *=* ΔΦ = SΔ*T*. The open-circuit Seebeck voltage of the device is directly measured (Keithley Source Meter 2612).

The micro-Raman thermometry measurements are conducted using the HORIBA LabRAM HR800 system. A 633-nm He-Ne laser is focused by a Nikon 50x long-working-distance objective lens, at the contact to obtain maximum Seebeck voltage signal. The focused laser locally heats up the sample and induces a Raman scattering shift which is collected and sent to the HORIBA LabRAM HR800 (1800 groove/mm grating). The temperature-dependent Raman shift of E_g_^2^ mode is calibrated as the temperature transducer (χ_θ_=0.015cm^-1^/K)^2^.

A numerical model consists of laser heating, heat conduction, and thermoelectrics (including electrical conduction, Seebeck/Peltier effect, and Joule heating) is built for both Seebeck coefficient procedure discussed in the main text and the validation here. The properties of Bi_2_Te_2_Se are included in all the physical models. The contact metal is included in all other processes except for the Seebeck effect due to the small Seebeck coefficient and small temperature gradient. The SiO_2_/Si substrate and ambient air is only considered in the heat transfer model.

The temperature rise for the validation is also calculated in this model using open circuit electrical boundary condition instead of the short-circuit condition used in the main text, and is compared to the measured temperature rise using micro-Raman thermometry. Figure S1 shows details of the Raman thermometry measurement. At a given incident power, the Raman shift of the E_g_^2^ mode is measured to find the temperature rise, and is compared with the heat transfer modeling result (Fig. S1a). Figure S1b shows the measured open-circuit voltage vs incident power and the temperature rise. The temperature rise obtained from modeling is lower than that measured using Raman thermometry by about 15%, mainly due to the errors propagating from optical properties and thermal conductivity measurements. This systematic error is corrected in the Seebeck coefficient in the main text (Figure 2).

**Figure S1.** Extracting Seebeck coefficient from temperature rise and thermoelectric voltage. (a) Temperature rise under varied incident laser power by heat transfer modeling and Raman thermometry measurement. (b) Temperature rise vs. laser power when laser spot at a contact and the corresponding open circuit Seebeck voltages.

**Supplementary Note 2.** Estimation of the Fermi level and depletion layer thickness under gating

The shift of Fermi level under a certain Vg can be estimated using the depletion mode MOS capacitance model^3^.

$$\begin{aligned} V_{g}=\psi_{s}+\frac{\varepsilon_{body}}{\varepsilon_{ox}}t_{ox}\sqrt{\frac{2eN}{\varepsilon_{body}}\psi_{s}}\#\left( S1.1 \right) \end{aligned}$$

$$\begin{aligned} W_{d}=\sqrt{\frac{\varepsilon_{body}\psi_{s}}{eN}}\#\left( S1.2 \right) \end{aligned}$$

Here, *ψ*_s_ is the surface potential, representing the Fermi level shift; ε is the dielectric constants for oxide layer (ox) and the channel material Bi_2_Te_2_Se (body), respectively; *e* is the electron charge, *N* is the bulk carrier concentration; and *W*_d_ is the depletion layer thickness.

Solving the two equations using $\varepsilon_{body}$ ~ 113 of Bi_2_Se_3_^4^ and *N* ~ 2×10^12^ cm^-2^ extracted from Hall effect measurements of an 11-nm and a 16-nm Bi_2_Te_2_Se device^2^, under 20 V gating, the depletion depth is found to be ~32 nm and the bottom Fermi level is shifted by ~0.5 eV. It is noted that the estimated Fermi level shifting is approximate. When the Fermi level reaches the vicinity of the band edge a different model needs to be used to estimate the Fermi level shifting.

**Supplementary Note 3.** Calculation of Seebeck coefficient using Landauer formalism

Landauer formalism is an effective framework to evaluate transport properties in terms of the transmission of states. Here we adopt Landauer formalism to estimate the Seebeck coefficient using minimum knowledge of the dimensionality, electronic dispersion, and Fermi level^5–7^. The potential and temperature-driven electrical charge transport can be written as^7,8^,

$$\begin{aligned} j_{e}=\mathcal{L}_{11}\left( -\frac{d\Phi}{\mathrm{dx}} \right)+\mathcal{L}_{12}\left( -\frac{dT}{\mathrm{dx}} \right)\#\left( S2.1 \right) \end{aligned}$$

$$\begin{aligned} \mathcal{L}_{11}=\frac{e^{2}}{3}\int v^{2}\tau\frac{\partial f}{\partial E}D\left( E \right)dE \#\left( S2.2 \right) \end{aligned}$$

$$\begin{aligned} \mathcal{L}_{12}=\frac{e}{T}\int v^{2}\tau\left( E-E_{F} \right)\frac{\partial f}{\partial E}D\left( E \right)dE\#\left( S2.3 \right) \end{aligned}$$

$$\begin{aligned} S=\frac{\mathcal{L}_{12}}{\mathcal{L}_{11}}\#\left( S2.4 \right) \end{aligned}$$

Where *j*_e_ is the electrical current density, *v* is the group velocity, τ is the relaxation time, *f* is the Fermi function, *E*_F_ is the Fermi level, and *D*(E) is the density of states (DOS) as a function of the energy. The absolute value of Seebeck coefficient is used for brevity in the discussion below, unless specified. For given dimensionality (e.g., 3D bulk state or 2D surface state) and dispersion (e.g. parabolic for most single band semiconductors, linear for Dirac cone systems, etc.), the density of states and group velocity can be directly obtained, and the scattering rate (1/τ) can be assumed to be proportional to the density of states. Furthermore, the details of effective mass, degeneracies, etc. in *L*_11_ and *L*_12_ are canceled out so that for semiconductors with the same type of dispersion and dimensionality, the Seebeck coefficient at a given Fermi level is the same. For example, for 3D electrons with parabolic dispersion, DOS ~ (*E*-*E*_c_)^1/2^ and *v* ~ (*E*-*E*_c_) so that the Seebeck coefficient is^6^

$$\begin{aligned} S_{3D,para}=-\frac{k_{B}}{e}\left[ \frac{\left( \frac{5}{2} \right)\mathcal{F}_{\frac{3}{2}}\left( \eta_{F,C} \right)}{\mathcal{F}_{\frac{1}{2}}\left( \eta_{F,C} \right)}-\eta_{F,C} \right]\#\left( S3 \right) \end{aligned}$$

In which k_B_ is the Boltzmann constant, *η*_F,C_ = (*E*_F_-*E*_c_)/ k_B_*T* is the normalized Fermi level at temperature *T*, with respect to the conduction band edge, and *F*_i_ is the i-th order Fermi integral.

Similarly, for the TSS with 2D surface states and linear dispersion, DOS is linearly dependent on (*E*-*E*_D_) and group velocity is constant, the Seebeck coefficient can be estimated by,

$$\begin{aligned} S_{2D,linear}=-\frac{k_{B}}{e}\left[ \frac{3\mathcal{F}_{2}\left( \eta_{F,D} \right)}{\mathcal{F}_{1}\left( \eta_{F,D} \right)}-\eta_{F,D} \right]\#\left( S4 \right) \end{aligned}$$

Notice here *η*_F,D_ = (*E*_F_-*E*_D_)/ k_B_*T* is the normalized Fermi level from the Dirac point. The Seebeck coefficients for bulk conduction band and TSS are calculated by numerical integration and plotted against the normalized Fermi level in Figure S2. Similar to calculations in Ref. ^5^, the Seebeck coefficient generally decreases with normalized Fermi level for both 3D and 2D electrons. The Fermi level is located near the mid-band gap since the carrier density at zero gating is very low. The measurement shows that the Seebeck coefficient is 120 μV/K. From Figure S2, this Seebeck coefficient corresponds to that of a 2D gas with a Fermi level near the band center. Figure S2 shows that if we consider a wide range of possible Fermi level, there is a clear separation between 160 and 220 μV/K for contributions from 2D TSS and 3D bulk. Therefore, the measured Seebeck coefficient cannot be a result of 3D bulk, but must be a result of 2D TSS.


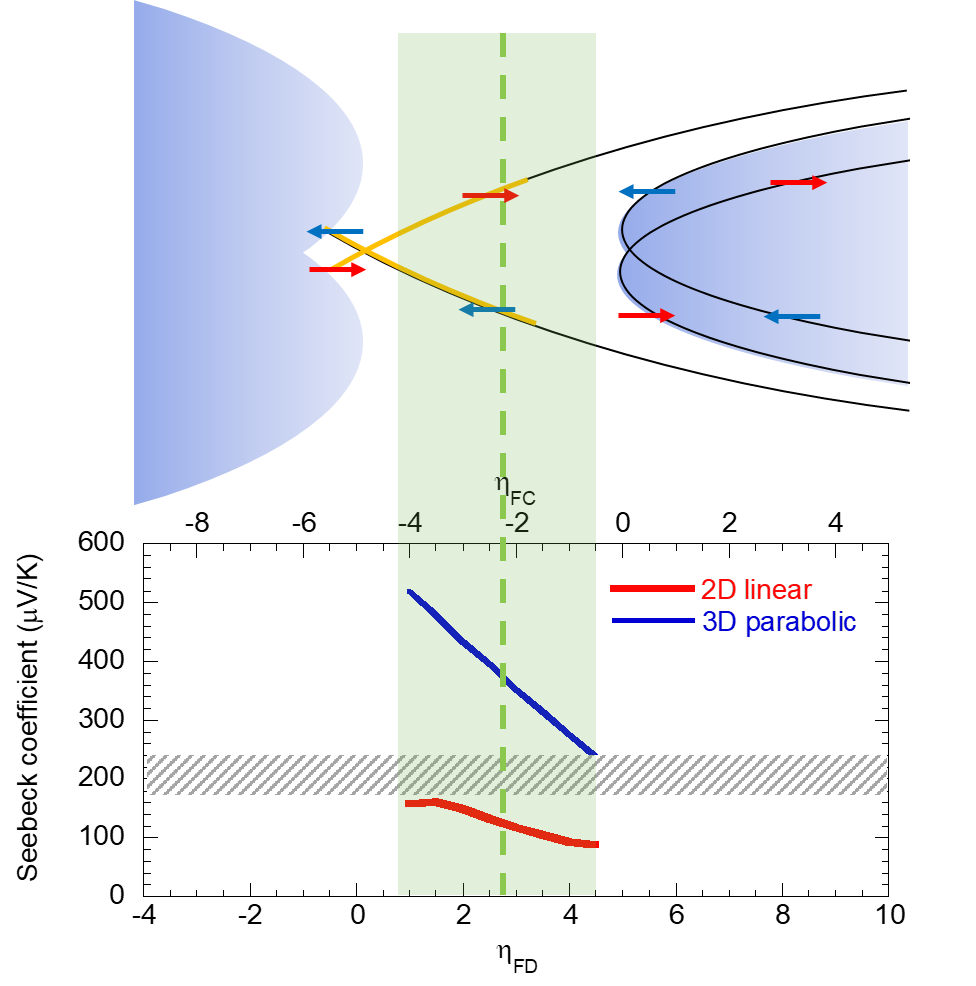


**Figure. S2.** Seebeck coefficient of bulk conduction band and topological surface states at different normalized Fermi level, calculated by Landauer formalism. The green dashed line indicates the intrinsic Fermi level near the middle of the bandgap, and the green region indicates a wider possible range of the Fermi level.

**Supplementary Note 4.** Origin of the linear-polarization dependent sin(4α) and cos(4α) photocurrent

The sin(4α) and cos(4α) terms obtained from the photocurrent measurement by rotating the quarter waveplate has been observed in previous photocurrent measurements in similar material systems. These measurements showed that, in addition to the galvanic current driven by circular polarization, there are also responses of certain types of carriers to the rotation of linear polarization. Two possible mechanisms have been proposed, namely the linear photogalvanic effect (direction of current depends on electrical field direction only) and the photon drag effects (direction of current depends on both electrical field and Poynting vector directions)^9–11^.

A simple treatment is adopted to handle both effects, considering only the second-order electrical field effect is linear within the measuring range of incident power^12^:

$\begin{aligned} j_{\lambda}=\sum_{\mu, \nu} \chi_{\lambda\mu\nu}E_{\mu}E_{\nu}^{*}+\sum_{\mu, \nu,\xi} \psi_{\lambda\mu\nu\xi}E_{\mu}E_{\nu}^{*}q_{\xi}+o\left( E^{2+} \right)\#\left( S5 \right) \end{aligned}$where $j(\vec{r}, t)$ is the electrical current density and$E(\vec{r}, t)$ is the electrical field. The expansion coefficients χ_λμν_, and *ψ*_λμνξ_ are 3^rd^ and 4^th^ rank tensor, respectively, describing the lattice structure and orientation. *E*^*^ is the complex conjugate of the *E* vector. *q* is the momentum vector of the incident light. Thus, the first term represents the photogalvanic effect that depends only on the electrical field direction, and the second term represents the photon drag effect that also depends on the direction of the wave vector of the incident light. For oblique incidence, Eq. (1) can be expressed as^10^,

$$\begin{aligned} \left[ \begin{matrix} j_{x} \\ j_{y} \end{matrix} \right]=\left[ \begin{matrix} -\cos\left( 2\alpha-2\phi\right)\left( \chi-T_{z}q+\frac{T_{\parallel}q\theta^{2}}{2} \right)t^{2}E_{0}^{2} \\ \sin\left( 2\alpha-2\phi\right)\left( \chi-T_{z}q+\frac{T_{\parallel}q\theta^{2}}{2} \right)t^{2}E_{0}^{2} \end{matrix} \right]\#\left( S6 \right) \end{aligned}$$

The origin of the linear polarization-dependent photocurrent is studied by rotating the device by 180 degrees while keeping the optical setup unchanged, so that the projection of the wavevector is reversed while the in-plane electromagnetic field magnitude remains the same. Here we repeat the photocurrent measurement with a half waveplate (HWP) to rotate the polarization direction. The linear polarization at HWP angle = 0 is also perpendicular to the channel. As shown in Figure S3, the variations of photocurrent vs. HWP angle are the same, showing the contribution from the linear photogalvanic effect, i.e. the dependence on the incident light polarization alignment with the lattice orientation. The discrepancy of the absolute value is inevitable due to the difficulty in aligning the laser focal spot at the exact same location. The photon drag effect plays a smaller role. This result also agrees with the microscale origin^10^ for which the photon drag requires a much larger sample to pronounce. In fact, all the reported observations of dominant photon drag currents so far are in bulk or chip-sized epitaxial samples.^10,13^

**Figure S3.** Photocurrent varying with polarization of light controlled by half-waveplate, oblique incident from two opposite direction (forward / backward).

The linear photogalvanic effect can be attributed to the angle of the lattice orientation with respect to in-plane electromagnetic oscillation. The classical treatment above requires the lattice to have a broken inversion symmetry so that the linear photogalvanic effect, i.e. the χ term to be finite. For the Bi_2_Te_2_Se lattice we studied, the bulk follows a D3d group preserving the inversion symmetry while its surface is described by the C3v group which lacks inversion symmetry. This means the L1 and L2 terms originate from the surface state. Generally speaking, the second-order effects result from the redistribution carriers in k-space under optical excitation, instead of polarization of the matter. The DC current originated from the optical oscillating field can be microscopically explained by the asymmetric scattering of the carriers by the inversion-broken lattice potential. As shown in Figure S4 below, for the lattice (bulk) preserving inversion symmetry (left), the scattering is symmetric resulting in no net current. However, in an inversion-symmetry-breaking lattice (surface), carriers may lose more momentum when scattered with the lattice potential in inversed direction, accumulated to generate a net current flow^9^.

**Figure S4.** Microscopic picture of the linear photogalvanic effect, of the bulk (left) inversion symmetry preserving states (e.g. C6v group), and surface (center and right) inversion-symmetry-breaking states (C3v group). The black double-headed arrows denote the polarization of the incident light, the blue arrows denote the net flow of the majority carriers. **Supplementary Note 5.** Tunability of photocurrent in thicker Bi_2_Te_2_Se devices

Two thicker devices (27-nm and 74-nm) are also fabricated and studied using the same approach. The results are shown in Figure S5. Noticed that the majority carrier type is p-type for the much thicker 74-nm device, which can be clearly observed from the field-effect transport curve (Figure S5a) and the photo-thermoelectric pattern (Figure S5b). Now that the bulk carriers are dominant in the 74-nm Bi_2_Te_2_Se film, the intrinsic Fermi level is close to the valence band, which agrees with our previous Hall effect measurement for thick film and bulk Bi_2_Te_2_Se^2,14^.

The Seebeck coefficients are also extracted for the 27-nm and 74-nm devices (Figure S5c), ~ -300 and 400 μV/K respectively. Based on the analysis in Supplementary Note 3, the dominant carriers are from the bulk band. In the 27-nm film, the band-bending due to surface oxidation (shown by ARPES^14^) is expect to form an inversion layer with conduction band electrons and show an averaged n-type behavior in the entire film. From the results observed in devices with different thicknesses, the surface band-bending plays an important role that affect which type of carrier dominate. Thus, special precautions, including but not limited to thickness control^2,15^ and surface chemistry^16,17^, are needed in fabricating tetradymite-based TI devices with desired performance.

As shown in Figure S5d, in the 74-nm film, the abundant bulk carriers screen the top TSS from the back gating and the helical photocurrent is not tuned effectively. For the 27-nm device, there is some observable tuning when positive gating brings the Fermi level to the TSS warping region but helicity-control is less effective compared to the 11-nm film shown in the main text, and back gating is not sufficient to turn off the thermoelectric effect completely.

**Figure S5.** Field-effect tunability of helical and photo-thermoelectric current in 27-nm and 74-nm Bi_2_Te_2_Se films. (a) Sheet conductance at varied gate voltage. (b) Photocurrent of incident light vs. focal spot location along the channel. (c) Seebeck coefficient extracted using the polarization insensitive component of the photocurrent. (d) Helicity-tuned Seebeck coefficient extracted using the helical photocurrent.

**References**

1. Luo, Z. *et al.* Measurement of In-Plane Thermal Conductivity of Ultrathin Films Using Micro-Raman Spectroscopy. *Nanoscale Microscale Thermophys. Eng.* **18**, 183–193 (2014) , doi:10.1080/15567265.2014.892553.

2. Luo, Z. *et al.* Large Enhancement of Thermal Conductivity and Lorenz Number in Topological Insulator Thin Films. *ACS Nano* **12**, 1120–1127 (2018) , doi:10.1021/acsnano.7b06430.

3. Sze, S. M. & Ng, K. K. *Physics of Semiconductor Devices*. *Physics of Semiconductor Devices* (John Wiley & Sons, Inc., 2006). , doi:10.1002/0470068329.

4. Madelung, O., Rössler, U. & Schulz, M. *Non-Tetrahedrally Bonded Elements and Binary Compounds I*. *Non-Tetrahedrally Bonded Elements and Binary Compounds I* (Springer-Verlag, 1998). , doi:10.1007/b71138.

5. Kim, R., Datta, S. & Lundstrom, M. S. Influence of dimensionality on thermoelectric device performance. *J. Appl. Phys.* **105**, 034506 (2009) , doi:10.1063/1.3074347.

6. Jeong, C., Kim, R., Luisier, M., Datta, S. & Lundstrom, M. On Landauer versus Boltzmann and full band versus effective mass evaluation of thermoelectric transport coefficients. *J. Appl. Phys.* **107**, 023707 (2010) , doi:10.1063/1.3291120.

7. Lundstrom, M. & Jeong, C. Near-equilibrium Transport: Measurements. in *Near-Equilibrium Transport* 143–168 (WORLD SCIENTIFIC, 2013). , doi:10.1142/9789814329873_0008.

8. Imry, Y. & Landauer, R. Conductance viewed as transmission. *Rev. Mod. Phys.* **71**, S306 (1999) , doi:10.1103/revmodphys.71.s306.

9. Olbrich, P. *et al.* Room-temperature high-frequency transport of Dirac fermions in epitaxially grown Sb_2_Te_3_ - And Bi_2_Te_3_ -based topological insulators. *Phys. Rev. Lett.* **113**, 1–5 (2014) , doi:10.1103/PhysRevLett.113.096601.

10. Plank, H. *et al.* Photon drag effect in (Bi1-xSbx)2Te3 three-dimensional topological insulators. *Phys. Rev. B* **93**, 125434 (2016) , doi:10.1103/PhysRevB.93.125434.

11. Plank, H. *et al.* Opto-electronic characterization of three dimensional topological insulators. *J. Appl. Phys.* **120**, 165301 (2016) , doi:10.1063/1.4965962.

12. Glazov, M. M. & Ganichev, S. D. High frequency electric field induced nonlinear effects in graphene. *Physics Reports* vol. 535 101–138 (2014) , doi:10.1016/j.physrep.2013.10.003.

13. Ganichev, S. & Prettl, W. *Intense Terahertz Excitation of Semiconductors*. *Intense Terahertz Excitation of Semiconductors* (Oxford University Press, 2007). , doi:10.1093/acprof:oso/9780198528302.001.0001.

14. Cao, H. *et al.* Controlling and distinguishing electronic transport of topological and trivial surface states in a topological insulator. *arXiv Prepr. arXiv …* **1409.3217**, 1–27 (2014).

15. Pettes, M. T., Maassen, J., Jo, I., Lundstrom, M. S. & Shi, L. Effects of surface band bending and scattering on thermoelectric transport in suspended bismuth telluride nanoplates. *Nano Lett.* **13**, 5316–5322 (2013) , doi:10.1021/nl402828s.

16. Walsh, L. A. *et al.* Interface Chemistry of Contact Metals and Ferromagnets on the Topological Insulator Bi _2_ Se _3_. *J. Phys. Chem. C* **121**, 23551–23563 (2017) , doi:10.1021/acs.jpcc.7b08480.

17. Wang, E. *et al.* Robust Gapless Surface State and Rashba-Splitting Bands upon Surface Deposition of Magnetic Cr on Bi _2_ Se _3_. *Nano Lett.* **15**, 2031–2036 (2015) , doi:10.1021/nl504900s.
